# Evaluating an AI-assisted triage workflow for retinal diseases

**DOI:** 10.1371/journal.pdig.0001700

**Published:** 2026-09-15

**Authors:** Doohyun Park, Richul Oh, Jihyeon Baek, Kunho Bae

**Affiliations:** 1 VUNO Inc., Seoul, Korea; 2 Department of Ophthalmology, Seoul National University Hospital, Seoul, Korea; 3 Mass Eye and Ear and the Department of Ophthalmology at Harvard Medical School, Boston, Massachusetts, United States of America; London School of Hygiene & Tropical Medicine, UNITED KINGDOM OF GREAT BRITAIN AND NORTHERN IRELAND

## Abstract

The clinical value of artificial intelligence (AI) in fundus photography depends on workflow efficiency as well as diagnostic performance. We evaluated an AI-assisted negative-screening workflow for diabetic retinopathy (DR), retinal vein occlusion (RVO), and age-related macular degeneration (AMD) using 6,904 color fundus photographs independently graded by three retina specialists. In this workflow, AI-negative images were classified as negative without ophthalmologist review, whereas AI-positive images were referred for human interpretation. For each reader–disease pair, the reference standard was agreement between the other two readers; discordant cases were excluded (DR, 1.2%–2.3%; RVO, 0.3%–0.6%; AMD, 6.1%–11.6%). The workflow reduced direct ophthalmologist review to 7.3%–8.0% of images for DR, 11.7%–11.9% for RVO, and 13.5%–16.8% for AMD. For DR, sensitivity decreased significantly (0.9168 to 0.8838; difference, -0.0330; 95% confidence interval [CI], -0.0495 to -0.0165; *p* < 0.001), whereas specificity did not change significantly (0.9961 to 0.9983; *p* = 0.183). For RVO, sensitivity was unchanged (0.9807) and specificity did not change significantly (0.9985 to 0.9988; *p* = 0.320). For AMD, sensitivity decreased significantly (0.8570 to 0.8445; difference, -0.0124; 95% CI, -0.0215 to -0.0033; *p* = 0.008), whereas specificity did not change significantly (0.9685 to 0.9843; *p* = 0.324). In an exploratory image-level analysis for at least one target disease, review decreased to 27.7%–30.1%; sensitivity changed from 0.9122 to 0.9039 (difference, -0.0083; 95% CI, -0.0131 to -0.0035; *p* < 0.001) and specificity from 0.9659 to 0.9804 (*p* = 0.334). AI-assisted negative-screening can reduce ophthalmologist workload across multiple retinal diseases, but with disease-specific safety trade-offs: because AI-negative images are not reviewed, reduced sensitivity for DR and AMD means a small proportion of true-positive cases would go undetected, potentially delaying diagnosis and treatment for sight-threatening disease. These results support disease-specific implementation strategies that balance workload reduction against missed positive cases.

## Author summary

Screening retinal photographs for common eye diseases is important, but reviewing every image requires specialist time. Many studies of artificial intelligence (AI) ask whether software can diagnose diseases on its own. We asked a different question: can it make screening more efficient when used before an ophthalmologist reviews an image? We tested a workflow in which AI software first examined 6,904 retinal photographs for diabetic retinopathy (DR), retinal vein occlusion (RVO), and age-related macular degeneration (AMD). Images classified as negative were not reviewed further, while positive cases were referred to an ophthalmologist. This approach reduced direct specialist review to about 7%–8% of images for diabetic retinopathy, about 12% for retinal vein occlusion, and about 14%–17% for age-related macular degeneration. For RVO, performance was essentially unchanged compared with human-only reading. For DR and AMD, the workflow missed slightly more positive cases, with improved or unchanged specificity. Overall, these findings suggest that AI may be most useful as a triage tool in retinal screening, helping specialists focus on higher-risk images while balancing disease-specific trade-offs.

## 1 Introduction

Artificial intelligence (AI) has rapidly advanced the analysis of color fundus photographs (CFPs), with numerous studies demonstrating that deep learning algorithms can detect major retinal diseases with accuracy comparable to human experts [1,2]. However, the deployment of fully autonomous AI for primary diagnosis remains a complex and sensitive challenge in real-world clinical settings [3,4]. Beyond technical performance, autonomous interpretation without human oversight raises unresolved concerns regarding accountability, legal liability, and regulatory acceptance [5]. As a result, despite strong evidence of technical feasibility, the widespread adoption of AI as a standalone diagnostician remains limited [5,6].

Given these constraints, the focus of AI implementation is increasingly shifting toward strategies that integrate AI into existing clinical workflows [7,8]. In high-throughput screening environments, where the burden of retinal disease often exceeds specialist capacity, the most immediate value of AI lies not in replacing clinicians but in optimizing their workflow [7,9]. In this context, AI-based triage or filtering approaches—such as negative-screening workflows, in which AI-negative images are accepted as negative without further review while AI-positive images proceed to ophthalmologist interpretation—offer a practical pathway to improve efficiency. Unlike autonomous referral, in which the AI issues the final referral decision, this design retains the ophthalmologist as the sole diagnostician for all AI-positive images and uses AI only to remove likely normal images from the review queue. Nonetheless, because AI-negative images are not reviewed, any false-negative AI decision becomes a missed positive, so efficiency gains must be weighed against disease-specific safety and confirmed prospectively.

Despite this need, workflow-oriented evidence remains relatively limited compared with the extensive literature on standalone diagnostic performance [1,7]. Prior implementation studies have largely focused on single-disease settings, most commonly diabetic retinopathy (DR), and have more often evaluated autonomous referral or AI-assisted interpretation rather than negative-screening strategies [8,10]. Evidence is particularly limited for multi-disease workflows in which AI triage is combined with continued human interpretation across a defined set of major retinal diseases.

To address this gap between technological capability and clinical implementation, we evaluated an AI-assisted negative-screening workflow for CFP interpretation in a multi-disease setting involving three common and clinically significant targets: DR, retinal vein occlusion (RVO), and age-related macular degeneration (AMD). By retrospectively simulating this triage approach across multi-reader multi-case scenarios, we aimed to quantify the reduction in ophthalmologist review burden and to assess the associated diagnostic trade-offs at the workflow level. The present study is distinguished by three features: integration of a negative-screening workflow across multiple retinal diseases; prespecified disease-specific multi-reader multi-case (MRMC) comparisons as the primary analysis, complemented by generalized linear mixed models (GLMM); and explicit quantification of disease-specific trade-offs between reduced direct ophthalmologist review and changes in diagnostic sensitivity.

## 2 Materials and methods

### 2.1 Ethics statement

The study was approved by the Institutional Review Board of the Seoul National University Hospital (SNUH) (IRB No. 2402-005-1506). As the study utilized a retrospective dataset of de-identified fundus photographs, the requirement for obtaining written informed consent from patients was waived by the IRB.

### 2.2 Study design and dataset

The dataset comprised 6,904 gradable CFPs obtained from 2,593 patients at Seoul National University Hospital (SNUH) between 2004 and 2024, after exclusion of poor-quality or ungradable images during dataset construction. This study cohort was independent of the datasets used for AI development, including model training and operating-threshold selection. CFPs were acquired using multiple fundus cameras, including the VISUCAM 524 (Carl Zeiss Meditec AG), TRC-50DX/TRC-50DX Type IA (Topcon Corporation), and VX-10α and nonmyd 7 (Kowa Company, Ltd.), under mixed mydriatic and nonmydriatic conditions.

Each CFP was independently reviewed by three board-certified ophthalmologists specializing in retina (R1, R2, and R3), with 11, 10, and 7 years of clinical experience in retinal diseases, respectively. The readers were masked to one another’s assessments and to the AI outputs during annotation. For each image, they assigned binary labels for 12 finding-level endpoints and 3 disease-level endpoints, following previous studies [11,12].

**Fig 1:**
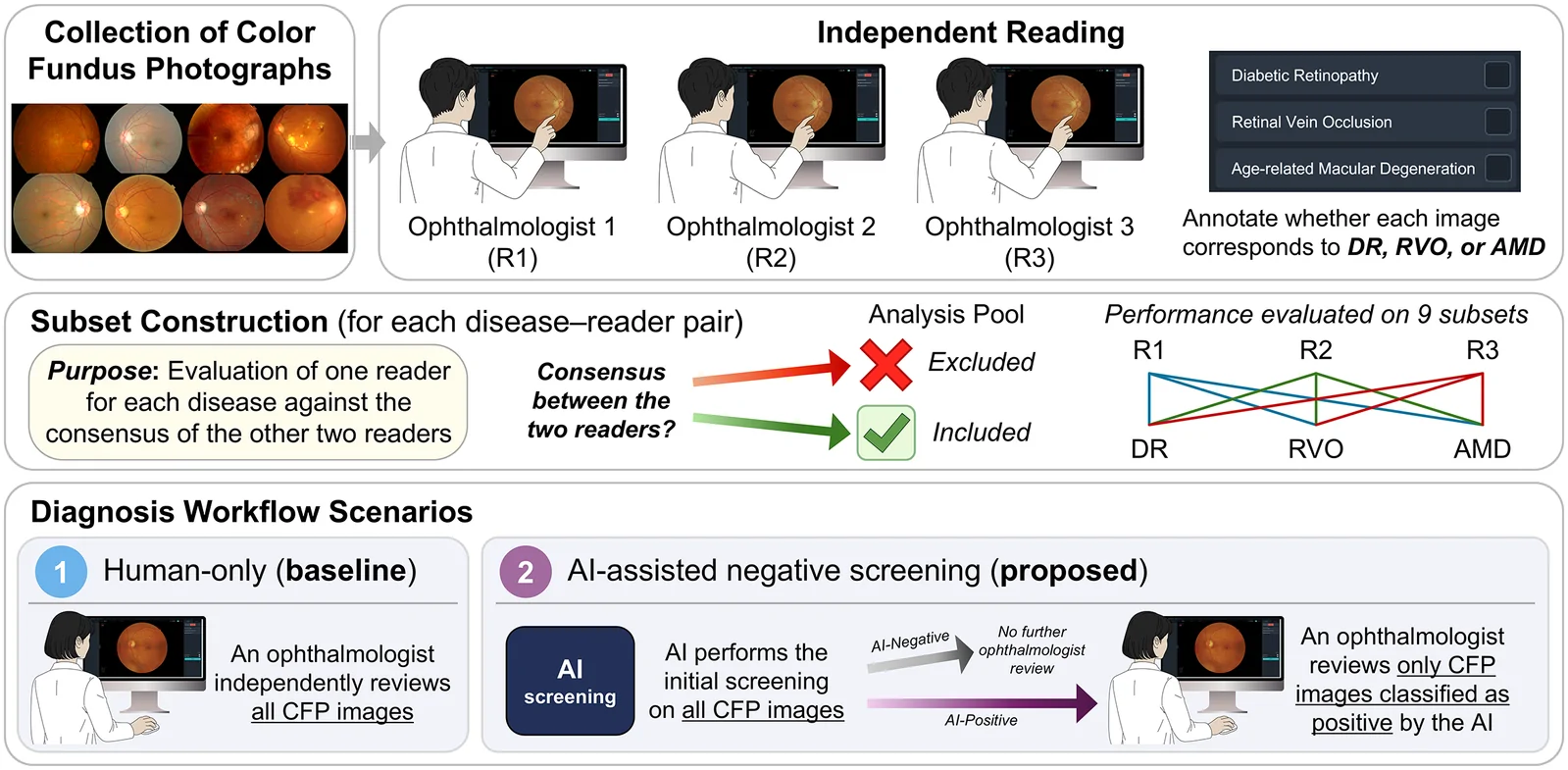
Study design and pairwise reader–disease evaluation framework for the AI-assisted workflow. Three ophthalmologists independently reviewed each color fundus photograph (CFP) for diabetic retinopathy (DR), retinal vein occlusion (RVO), or age-related macular degeneration (AMD). For each reader–disease pair, one reader was evaluated against the agreement of the other two, with discordant reference cases excluded. Human-only interpretation was compared with an AI-assisted negative-screening workflow, in which only AI-positive cases underwent ophthalmologist review.

In this study, DR was defined as moderate nonproliferative diabetic retinopathy (NPDR) or worse and/or diabetic macular edema (DME), corresponding to referral-warranted disease [13]. CFPs graded as normal, mild DR, or retinal abnormalities unrelated to DR were classified as non-DR. RVO was defined by fundus findings compatible with either central retinal vein occlusion (CRVO) or branch retinal vein occlusion (BRVO) [14]; eyes not meeting these criteria were classified as non-RVO. AMD was graded according to established diagnostic criteria. AMD-positive cases were defined as eyes with early, intermediate, or late AMD, whereas eyes without AMD were classified as no AMD [15]. The overall study design is illustrated in Fig 1.

### 2.3 Reference standard construction

A leave-one-out consensus approach was used to construct the reference standard and to reduce incorporation bias. For each reader under evaluation, the reference standard was defined by the agreement of the other two readers. Only cases for which the two non-index readers agreed on positive or negative status for the target disease were included. Because a discordant case could not be classified as either positive or negative for the target disease, no valid reference label could be assigned, and such cases were therefore excluded from the disease-specific analyses. Accordingly, the number of evaluable cases differed across reader–disease pairs.

For the image-level composite analysis, reference status was defined by agreement of the two non-index readers on the presence or absence of at least one target disease (DR, RVO, or AMD). An image was classified as reference-positive if both non-index readers agreed that at least one target disease was present. An image was classified as reference-negative only if both readers agreed that all three target diseases were evaluable and absent. Images without any positive label but with disagreement for at least one target disease were excluded from the composite analysis.

### 2.4 AI-assisted workflow

The conventional workflow consisted of human-only interpretation, in which the final classification of each evaluable case was determined solely by the ophthalmologist’s binary reading. The intervention was an AI-assisted negative-screening workflow. AI screening was performed using VUNO Med-FundusDx, an AI-based fundus image analysis software certified by the Ministry of Food and Drug Safety (MFDS), Republic of Korea. The target diseases in this study were limited to DR, RVO, and AMD, because these were the disease indications for which the software had received MFDS approval. Accordingly, other fundus-detectable conditions, such as glaucoma or epiretinal membrane, were not included in the present workflow evaluation. For each target disease, the AI system generated a prespecified binary screening result using a disease-specific operating threshold selected on a separate validation dataset to target a sensitivity of 0.99. These thresholds were fixed before the present analysis and were not tuned on the current study cohort.

Under the AI-assisted workflow, cases classified as AI-negative were assigned a final negative classification without ophthalmologist review, whereas cases classified as AI-positive were referred for ophthalmologist interpretation, and the ophthalmologist’s reading determined the final classification. For the image-level workflow assessing the presence of at least one target disease across DR, RVO, and AMD, AI triage was applied jointly across all three diseases. An image was referred for ophthalmologist review if the AI system screened positive for at least one target disease; otherwise, it was classified directly as negative. Among referred images, the final classification was positive if the ophthalmologist identified at least one target disease and negative otherwise.

Because all three readers had independently interpreted every image in the dataset, the AI-assisted workflow was evaluated by retrospectively simulating the AI triage decision on the original reader outputs. This paired design enabled within-reader comparisons between the conventional and AI-assisted workflows using identical case sets.

### 2.5 Study outcomes

#### 2.5.1 Primary endpoint.

The primary outcome was the between-workflow difference in disease-specific sensitivity and specificity for the detection of DR, RVO, and AMD. For each disease, both sensitivity and specificity were evaluated within an MRMC framework to account for variability attributable to both readers and cases.

#### 2.5.2 Secondary endpoints.

The secondary outcome was overall diagnostic accuracy, defined as the proportion of correct classifications, which was analyzed jointly across diseases and readers using a GLMM [16]. To quantify disease-specific workflow effects, an interaction model including workflow, disease, and a workflow-by-disease term was fitted, and disease-specific odds ratios (ORs) with 95% confidence intervals [CIs] were estimated from post hoc contrasts of estimated marginal means.

#### 2.5.3 Exploratory endpoints.

An exploratory composite outcome was defined as the detection of at least one target disease (DR, RVO, or AMD) versus no target disease. For this composite endpoint, sensitivity, specificity, and overall accuracy were compared between workflows using both MRMC and GLMM approaches.

### 2.6 Statistical analysis

#### 2.6.1 MRMC analysis (primary analysis).

The primary analysis used an MRMC framework based on the Obuchowski–Rockette method [17]. This approach estimates differences in diagnostic performance between workflows while accounting for the correlation structure introduced by multiple readers interpreting overlapping sets of cases. Both readers and cases were treated as random effects to allow generalization beyond the specific study sample.

Separate MRMC analyses were performed for sensitivity and specificity for each target disease (DR, RVO, and AMD), yielding six disease-specific comparisons between the AI-assisted and human-only workflows. For each analysis, the between-workflow difference and its corresponding 95% confidence interval (CI) were estimated.

#### 2.6.2 Generalized linear mixed models (secondary analyses).

GLMMs with a logit link and binomial error distribution were fitted as secondary analyses to evaluate diagnostic performance while accounting for clustering by reader and case. For overall diagnostic accuracy, fixed effects for workflow and disease were included, with random intercepts for reader and case.

To assess whether the effect of AI assistance differed by disease, an interaction model including a workflow-by-disease term was additionally fitted. This interaction model was applied to overall accuracy as well as to disease-specific sensitivity and specificity, the latter obtained by restricting analyses to disease-positive and disease-negative cases, respectively. Disease-specific effects of workflow were estimated using post hoc contrasts of estimated marginal means and are reported as ORs.

For the exploratory composite endpoint, disease-specific labels were collapsed into a single binary outcome (composite-positive vs. composite-negative). Composite accuracy was analyzed using a reduced GLMM including workflow as a fixed effect and reader and case as random intercepts. Composite sensitivity and specificity were then evaluated separately by restricting the analysis to composite-positive and composite-negative cases, respectively, using both GLMM and MRMC approaches. These analyses were considered exploratory and were interpreted cautiously.

For descriptive reader-level comparisons, performance metrics were calculated for each reader under both workflows. Within-reader differences in accuracy, sensitivity, and specificity were assessed using exact McNemar tests [18].

#### 2.6.3 Statistical inference and multiplicity.

Two-sided 95% CIs for reader-level and workflow-specific proportion-based performance measures were calculated using exact Clopper-Pearson methods based on the corresponding confusion-matrix counts. For MRMC comparisons, CIs were derived using the Obuchowski-Rockette method [17], and ORs from GLMMs are reported with Wald-type 95% CIs on the log-odds scale. All statistical analyses were performed in R (version 4.5.1; R Foundation for Statistical Computing, Vienna, Austria). MRMC analyses were conducted using the ‘MRMCaov’ package, and mixed-effects models were fitted using ‘lme4’. All tests were two-sided, and *p* < 0.05 was considered statistically significant.

Because multiple workflow comparisons were performed, formal inference was focused on the prespecified disease-specific MRMC comparisons of sensitivity and specificity. No formal multiplicity adjustment was applied; secondary GLMM analyses, composite endpoint analyses, threshold sensitivity analyses, and reader-level McNemar tests were interpreted as supportive, exploratory, or descriptive analyses, with emphasis placed on effect sizes and 95% CIs rather than isolated p values.

## 3 Results

### 3.1 Diagnostic performance by individual reader

Table 1 presents the number of evaluable cases for each reader–disease pair. The number of evaluable cases varied by disease because of the leave-one-out reference standard (DR: 6,746–6,819; RVO: 6,862–6,885; AMD: 6,101–6,481).

**Table 1:** Number of evaluable images for each reader–disease pair in the 6,904-image dataset.

Disease / Evaluated Reader	Reference Readers	Discordant cases (N)	Evaluable cases (N)	Reference positive (N)	Reference negative (N)
DR					
R1	R2, R3	137	6,767	436	6,331
R2	R1, R3	158	6,746	449	6,297
R3	R1, R2	85	6,819	482	6,337
RVO					
R1	R2, R3	19	6,885	312	6,573
R2	R1, R3	42	6,862	311	6,551
R3	R1, R2	35	6,869	310	6,559
AMD					
R1	R2, R3	423	6,481	560	5,921
R2	R1, R3	583	6,321	854	5,467
R3	R1, R2	803	6,101	588	5,513

DR, diabetic retinopathy; RVO, retinal vein occlusion; AMD, age-related macular degeneration.

Table 2 summarizes reader-level diagnostic performance under the human-only and AI-assisted workflows for DR, RVO, and AMD. The AI-assisted workflow markedly reduced the proportion of images requiring direct ophthalmologist review. Residual reader workload decreased to 7.3%–8.0% for DR, 11.7%–11.9% for RVO, and 13.5%–16.8% for AMD, corresponding to approximate reductions of 92%, 88%, and 85%, respectively.

**Table 2:** Detailed diagnostic performance by target disease and reader under the human-only and AI-assisted workflows.

Disease	Reader	N	Workflow	Workload, %	Accuracy(95% CI)	*p* ^†^	Sensitivity(95% CI)	*p* ^†^	Specificity(95% CI)	*p* ^†^
**DR**	R1	6,767	Human-only	100.0	0.9922 (0.9898–0.9941)	–	0.9564 (0.9328–0.9736)	–	0.9946 (0.9925–0.9963)	–
			AI-assisted	7.3	0.9931 (0.9908–0.9949)	0.405	0.9220 (0.8927–0.9454)	< 0.001	0.9979 (0.9965–0.9989)	< 0.001
	R2	6,746	Human-only	100.0	0.9953 (0.9933–0.9968)	–	0.9287 (0.9009–0.9507)	–	1.0000 (0.9994–1.0000)	–
			AI-assisted	7.3	0.9930 (0.9907–0.9949)	< 0.001	0.8953 (0.8632–0.9221)	< 0.001	1.0000 (0.9994–1.0000)	1.000
	R3	6,819	Human-only	100.0	0.9846 (0.9814–0.9874)	–	0.8651 (0.8314–0.8944)	–	0.9937 (0.9914–0.9955)	–
			AI-assisted	8.0	0.9855 (0.9824–0.9882)	0.405	0.8340 (0.7977–0.8661)	< 0.001	0.9970 (0.9953–0.9982)	< 0.001
**RVO**	R1	6,885	Human-only	100.0	0.9958 (0.9940–0.9972)	–	0.9776 (0.9543–0.9909)	–	0.9967 (0.9949–0.9979)	–
			AI-assisted	11.9	0.9965 (0.9948–0.9978)	0.062	0.9776 (0.9543–0.9909)	1.000	0.9974 (0.9959–0.9985)	0.062
	R2	6,862	Human-only	100.0	0.9991 (0.9981–0.9997)	–	0.9807 (0.9585–0.9929)	–	1.0000 (0.9994–1.0000)	–
			AI-assisted	11.7	0.9991 (0.9981–0.9997)	1.000	0.9807 (0.9585–0.9929)	1.000	1.0000 (0.9994–1.0000)	1.000
	R3	6,869	Human-only	100.0	0.9981 (0.9968–0.9990)	–	0.9839 (0.9628–0.9947)	–	0.9988 (0.9976–0.9995)	–
			AI-assisted	11.7	0.9983 (0.9970–0.9991)	1.000	0.9839 (0.9628–0.9947)	1.000	0.9989 (0.9978–0.9996)	1.000
**AMD**	R1	6,481	Human-only	100.0	0.9258 (0.9191–0.9320)	–	0.9857 (0.9720–0.9938)	–	0.9201 (0.9129–0.9269)	–
			AI-assisted	15.6	0.9614 (0.9564–0.9660)	< 0.001	0.9714 (0.9540–0.9836)	0.008	0.9605 (0.9552–0.9653)	< 0.001
	R2	6,321	Human-only	100.0	0.9492 (0.9435–0.9545)	–	0.6464 (0.6133–0.6785)	–	0.9965 (0.9946–0.9979)	–
			AI-assisted	16.8	0.9498 (0.9442–0.9551)	0.503	0.6370 (0.6037–0.6693)	0.008	0.9987 (0.9974–0.9995)	< 0.001
	R3	6,101	Human-only	100.0	0.9834 (0.9799–0.9865)	–	0.9388 (0.9162–0.9568)	–	0.9882 (0.9850–0.9909)	–
			AI-assisted	13.5	0.9867 (0.9835–0.9894)	0.001	0.9252 (0.9008–0.9451)	0.008	0.9933 (0.9908–0.9953)	< 0.001

^†^*P* values were calculated using exact McNemar tests. Workload indicates the proportion of cases requiring ophthalmologist review under each workflow. CI, confidence interval; DR, diabetic retinopathy; RVO, retinal vein occlusion; AMD, age-related macular degeneration.

**Fig 2:**
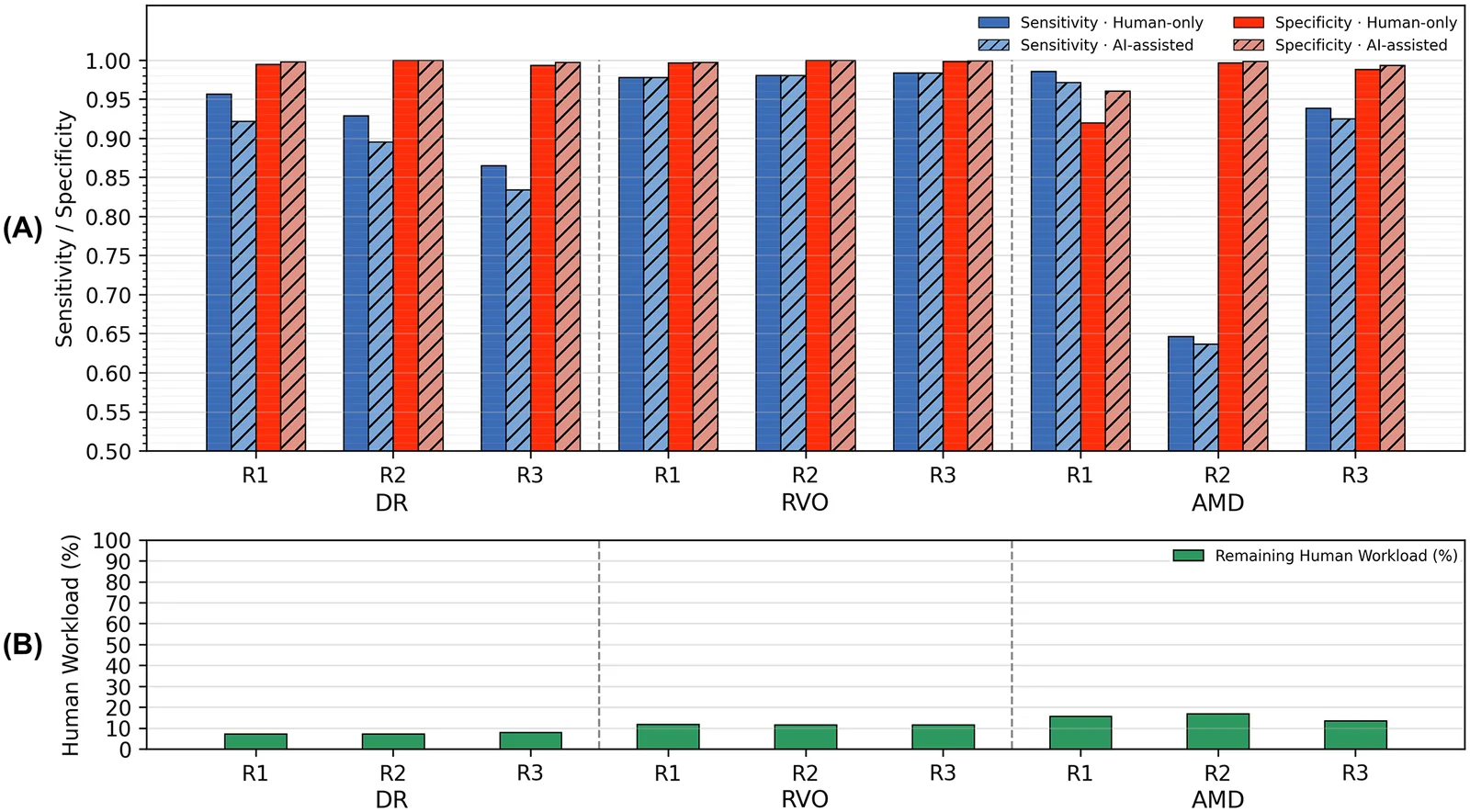
Performance across readers and target diseases under the human-only and AI-assisted workflows. **(A)** Sensitivity and specificity for each reader are shown for the human-only and AI-assisted workflows. **(B)** Human workload in the AI-assisted workflow, defined as the proportion of cases requiring ophthalmologist review. DR, diabetic retinopathy; RVO, retinal vein occlusion; AMD, age-related macular degeneration.

For DR and AMD, the AI-assisted workflow showed a consistent sensitivity–specificity trade-off, with significantly reduced sensitivity across all three readers for both diseases and significantly improved specificity in five of the six reader-level comparisons. Accuracy changes were reader-dependent, decreasing significantly only for Reader 2 in DR and improving significantly for Readers 1 and 3 in AMD. In contrast, RVO performance was preserved, with no significant differences in accuracy, sensitivity, or specificity between workflows. Detailed reader-level results are shown in Table 2 and Fig 2. For reference, the standalone diagnostic performance of the AI on the same pairwise evaluable datasets is provided in S1 Table. To assess the potential impact of excluding discordant cases, we additionally analyzed each discordant case under the assumption that it was either correctly or incorrectly classified; the resulting best-case and worst-case bounds on workflow performance are reported in S2 Table.

### 3.2 Disease-specific diagnostic performance

To evaluate disease-specific workflow effects while accounting for both reader and case variability, MRMC analyses were performed for sensitivity and specificity for each target disease (Table 3).

**Table 3:** Multi-reader multi-case (MRMC) comparison of diagnostic performance between the human-only and AI-assisted workflows by disease.

Disease	Metric	Human-only (Mean)	AI-assisted (Mean)	Difference(AI-assisted − Human-only)(95% CI)	*p* ^†^
DR	Sensitivity	0.9168	0.8838	-0.0330 (-0.0495 to -0.0165)	< 0.001
	Specificity	0.9961	0.9983	0.0022 (-0.0025–0.0070)	0.183
RVO	Sensitivity	0.9807	0.9807	0.0000 (-0.0000–0.0000)	1.000
	Specificity	0.9985	0.9988	0.0003 (-0.0007–0.0013)	0.320
AMD	Sensitivity	0.8570	0.8445	-0.0124 (-0.0215 to -0.0033)	0.008
	Specificity	0.9685	0.9843	0.0157 (-0.0364–0.0679)	0.324

^†^Statistical significance was assessed using the Obuchowski–Rockette model to account for reader and case variability. DR, diabetic retinopathy; RVO, retinal vein occlusion; AMD, age-related macular degeneration; CI, confidence interval.

For DR, mean sensitivity decreased from 0.9168 to 0.8838 (difference, -0.0330; 95% CI, -0.0495 to -0.0165; *p* < 0.001), whereas the increase in specificity did not reach statistical significance. For RVO, neither sensitivity nor specificity differed significantly between workflows. For AMD, the AI-assisted workflow was associated with a small but statistically significant reduction in sensitivity, from 0.8570 to 0.8445 (difference, -0.0124; 95% CI, -0.0215 to -0.0033; *p* = 0.008), whereas the increase in specificity did not reach statistical significance. Thus, the MRMC analysis showed a significant sensitivity trade-off for DR and AMD, with no statistically significant specificity gain, while RVO performance was preserved.

### 3.3 GLMM-based diagnostic performance

**Fig 3:**
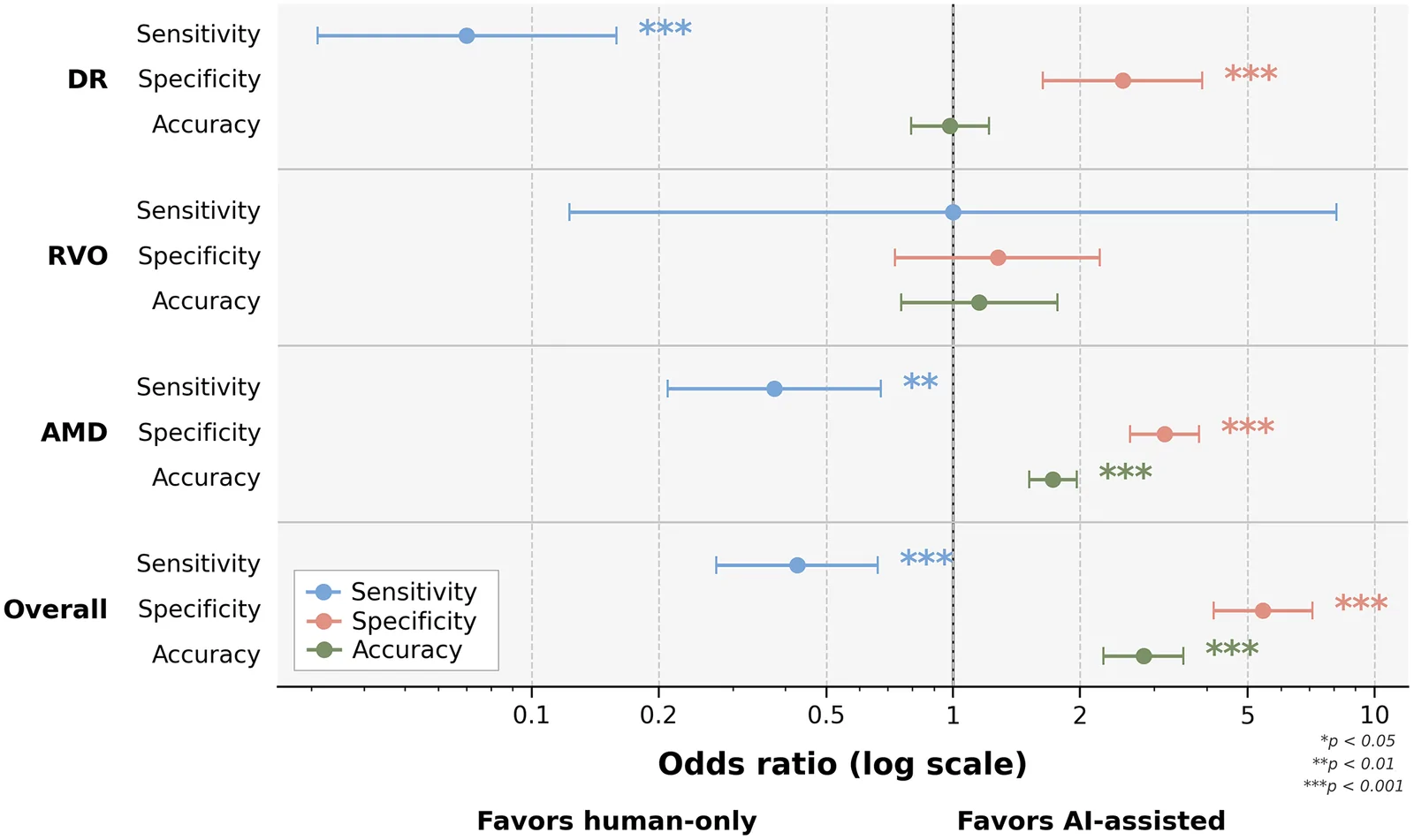
Generalized linear mixed model (GLMM)-derived odds ratios (ORs). DR, diabetic retinopathy; RVO, retinal vein occlusion; AMD, age-related macular degeneration. Asterisks indicate significance levels as follows: ^*^p < 0.05, ^**^p < 0.01, ^***^p < 0.001.

To quantify the relative effect of the AI-assisted workflow on the odds of correct classification, GLMMs with a workflow-by-disease interaction were fitted for sensitivity, specificity, and accuracy (Table 4; Fig 3).

**Table 4:** Generalized linear mixed model (GLMM)-derived odds ratios (ORs) for diagnostic performance under the AI-assisted workflow by disease.

Disease	Metric	AI-assisted (OR) (95% CI)	*p* ^†^
DR	Sensitivity	0.070 (0.031–0.159)	< 0.001
	Specificity	2.523 (1.634–3.896)	< 0.001
	Accuracy	0.983 (0.794–1.215)	0.871
RVO	Sensitivity	1.000 (0.123–8.122)	1.000
	Specificity	1.275 (0.728–2.230)	0.395
	Accuracy	1.153 (0.752–1.767)	0.514
AMD	Sensitivity	0.376 (0.210–0.673)	0.001
	Specificity	3.176 (2.631–3.834)	< 0.001
	Accuracy	1.725 (1.514–1.965)	< 0.001

Odds ratios (ORs) compare the AI-assisted workflow with the human-only workflow (reference = human-only). An OR > 1 indicates higher odds of correct diagnosis with AI assistance.

^†^Statistical significance was assessed using GLMMs. DR, diabetic retinopathy; RVO, retinal vein occlusion; AMD, age-related macular degeneration; CI, confidence interval.

For DR, the odds of correct classification differed significantly between workflows, with lower odds for sensitivity (OR, 0.070; *p* < 0.001) and higher odds for specificity (OR, 2.523; *p* < 0.001), whereas accuracy did not differ. For RVO, none of the differences reached statistical significance. For AMD, AI assistance was associated with lower odds of correctly classifying reference-positive cases (OR for sensitivity, 0.376; *p* = 0.001), higher odds of correctly classifying reference-negative cases (OR for specificity, 3.176; *p* < 0.001), and higher overall accuracy (OR, 1.725; *p* < 0.001).

### 3.4 Composite detection of at least one target disease

As an exploratory analysis, we evaluated a composite endpoint defined as the presence of at least one target disease (DR, RVO, or AMD) versus the absence of all three target diseases. This endpoint reflects a practical triage scenario in which the AI system determines whether a fundus photograph warrants further ophthalmologist review regardless of the specific disease label.

Reader-level results for the composite endpoint are summarized in Table 5. Under the AI-assisted workflow, the proportion of images requiring direct ophthalmologist review decreased to 27.7%–30.1% across readers, corresponding to an approximate 70%–72% reduction in review burden. Sensitivity decreased significantly for all three readers, although each decrease was only about 1% in absolute terms. For Readers 1 and 3, this decrease was offset by significant gains in specificity and accuracy, whereas Reader 2 showed no significant differences. As in the disease-specific analyses, inter-reader variability was most apparent for sensitivity.

**Table 5:** Reader-level performance for the composite endpoint of at least one target disease.

Metric	R1 (N = 6,419)			R2 (N = 6,304)			R3 (N = 6,124)		
	Human-only	AI-assisted	*p* ^†^	Human-only	AI-assisted	*p* ^†^	Human-only	AI-assisted	*p* ^†^
Workload	100.0%	28.8%		100.0%	30.1%		100.0%	27.7%	
Accuracy	0.9304 (0.9239–0.9365)	0.9581 (0.9529–0.9629)	< 0.001	0.9473 (0.9415–0.9527)	0.9461 (0.9402–0.9515)	0.077	0.9752 (0.9710–0.9789)	0.9773 (0.9733–0.9809)	0.047
Sensitivity	0.9932 (0.9872–0.9969)	0.9842 (0.9759–0.9902)	< 0.001	0.8038 (0.7837–0.8227)	0.7965 (0.7762–0.8157)	< 0.001	0.9395 (0.9257–0.9514)	0.9309 (0.9164–0.9436)	< 0.001
Specificity	0.9140 (0.9059–0.9215)	0.9513 (0.9450–0.9570)	< 0.001	0.9979 (0.9961–0.9990)	0.9987 (0.9972–0.9995)	0.125	0.9858 (0.9820–0.9890)	0.9911 (0.9880–0.9936)	< 0.001

Values in parentheses indicate 95% confidence intervals. ^†^*P* values for accuracy, sensitivity, and specificity were calculated using exact McNemar tests; Workload indicates the proportion of cases requiring ophthalmologist review under the corresponding workflow.

MRMC results for the composite endpoint are shown in Table 6. Composite sensitivity decreased slightly but significantly under the AI-assisted workflow (from 0.9122 to 0.9039; p < 0.001), whereas the increase in specificity (0.9659 to 0.9804) was not statistically significant.

**Table 6:** Multi-reader multi-case (MRMC) comparison for the composite endpoint (presence of at least one target disease).

Disease	Metric	Human-only (Mean)	AI-assisted (Mean)	Difference(AI-assisted − Human-only)(95% CI)	*p* ^†^
Composite endpoint	Sensitivity	0.9122	0.9039	-0.0083 (-0.0131 to -0.0035)	< 0.001
	Specificity	0.9659	0.9804	0.0145 (-0.0349–0.0639)	0.334

^†^Statistical significance was assessed using the Obuchowski–Rockette model to account for reader and case variability. CI, confidence interval.

GLMM results for the composite endpoint are summarized in Table 7 and Fig 3. AI assistance was associated with higher odds of correct classification for specificity (OR, 5.435) and overall accuracy (OR, 2.830), and significantly lower odds for sensitivity (OR, 0.427). Collectively, the composite analyses indicate that the AI-assisted workflow reduced direct review burden substantially, with improved specificity and accuracy but a sensitivity trade-off.

**Table 7:** Generalized linear mixed model (GLMM)-derived odds ratios for the composite endpoint of at least one target disease.

Metric	AI-assisted (OR) (95% CI)	*p* ^†^
Sensitivity	0.427 (0.274–0.663)	< 0.001
Specificity	5.435 (4.151–7.117)	< 0.001
Accuracy	2.830 (2.275–3.520)	< 0.001
Odds ratios (ORs) compare the AI-assisted workflow with the human-only workflow (reference = human-only). An OR > 1 indicates higher odds of correct classification with AI assistance. CI, confidence interval. ^†^Statistical significance was assessed using GLMMs.		

Odds ratios (ORs) compare the AI-assisted workflow with the human-only workflow (reference = human-only). An OR > 1 indicates higher odds of correct classification with AI assistance. CI, confidence interval. ^†^Statistical significance was assessed using GLMMs.

### 3.5 Threshold sensitivity analysis

Because a negative-screening workflow can be operated at different points on the sensitivity–workload curve, we compared the prespecified operating point (target sensitivity, 0.99) with a more conservative point (target sensitivity, 0.995) for each reader and disease (S3 and S4 Tables). At the 0.995 point, DR sensitivity rose from roughly 0.83–0.92 to 0.85–0.94 across readers, while residual review increased only modestly (about 7%–8% to 8%–9%). For AMD, the same change yielded little sensitivity gain but a larger increase in review burden (about 14%–17% to 18%–22%), and RVO sensitivity was unchanged. Within-reader comparisons against the human-only workflow at the 0.995 operating point remained statistically significant for DR and AMD and non-significant for RVO.

### 3.6 Post hoc review of false-negative cases

Among the 12 composite-level false-negative images, five were reference-labeled as DR and seven as AMD. Among the seven AMD-labeled cases, three demonstrated myopic patchy chorioretinal atrophy, two showed geographic atrophy, one showed neovascular AMD with concurrent peripapillary chorioretinal atrophy, and one showed advanced AMD with severe subretinal fibrosis. Among the five DR-labeled cases, three demonstrated moderate nonproliferative DR, including one image with an imaging artifact, one showed advanced DR with fibrovascular proliferation, and one showed regressed proliferative DR with prior laser photocoagulation scars.

## 4 Discussion

The present study showed that an AI-assisted negative-screening workflow substantially reduced direct ophthalmologist review burden across DR, RVO, and AMD, with varying effects on diagnostic performance across disease targets. Across reader-level, disease-level, and composite analyses, specificity generally improved under AI-assisted triage, whereas reductions in sensitivity were observed for DR, AMD, and the composite endpoint. Taken together, these findings suggest that AI-assisted triage may offer meaningful operational benefit in CFP interpretation, but its clinical acceptability is likely to be disease-specific and depends on whether any observed sensitivity loss is acceptable for the intended disease target and implementation setting.

A large body of work has established the technical feasibility of deep learning for fundus photograph–based diagnosis of DR, AMD, RVO, and multiple retinal abnormalities from CFPs, but most studies have evaluated AI primarily as a standalone classifier rather than as part of a clinical workflow [2,7,11,19–22]. Implementation-oriented studies have shown that AI can be deployed in several ways, including autonomous referral and hybrid teleophthalmology workflows; yet evidence remains limited for negative-screening designs in which AI excludes likely negative cases while retaining ophthalmologist interpretation for AI-positive images, particularly in multi-disease settings [3,8–10,23,24]. In that sense, the present work extends the literature by evaluating AI as a triage filter in nine concrete reader–disease scenarios and by examining its operating characteristics through both MRMC and GLMM frameworks in a setting where specialist reading capacity is an important operational constraint.

The disease-specific analyses showed that the operational value of the AI-assisted workflow was not uniform across targets. Residual workload was lowest for DR, intermediate for RVO, and highest for AMD. Across all three diseases, specificity generally increased under AI-assisted triage, but the magnitude and clinical implications of this gain differed by disease. For RVO, the primary MRMC analysis did not detect significant workflow-level differences in sensitivity or specificity, suggesting that the triage design largely preserved diagnostic performance while substantially reducing direct review burden. By contrast, for DR and AMD, the AI-assisted workflow was associated with a small but statistically significant reduction in sensitivity in the MRMC analysis, while specificity increased directionally and overall accuracy improved in the GLMM analysis. This pattern indicates that the balance between efficiency gain and missed positive cases is more constrained for DR and AMD than for RVO.

The higher residual workload and less favorable sensitivity profile for AMD are clinically plausible. Compared with RVO, AMD—particularly earlier dry AMD on CFPs—often involves more subjective grading thresholds and greater interpretive ambiguity, which may limit the efficiency of negative-screening [15,25]. This ambiguity is further reflected in substantial interobserver variability, even among experienced retina specialists [26]. In particular, early AMD often overlaps with age-related retinal changes, making it inherently challenging to determine whether such findings should be classified within the AMD spectrum [15,25]. Consistent with this, our study demonstrated marked variability in baseline sensitivity across readers, most notably for Reader 2 compared with the other two readers. In this context, the AI-assisted negative-screening workflow, despite a modest reduction in sensitivity, maintained high overall accuracy. These findings suggest that a predefined AI triage threshold can standardize the initial screening step before human interpretation, while preserving ophthalmologist review for AI-positive images.

While the primary MRMC analysis and the complementary GLMM analyses evaluate workflow performance from different statistical perspectives (absolute differences vs. log-odds scale), both approaches yielded consistent overall interpretations: RVO showed the greatest workload reduction without evidence of a sensitivity loss in the primary analysis, and DR and AMD showed the clearest trade-off between efficiency gain and reduced sensitivity. This trade-off is clinically important because, in the present negative-screening workflow, AI-negative images were classified directly as negative and were not reviewed by ophthalmologists; therefore, any false-negative triage decision constituted a missed positive case within the workflow itself. These findings support a disease-specific implementation perspective, in which the acceptable operating threshold should be determined not only by aggregate performance metrics but also by the clinical consequences of missed detection for each target disease.

To assess whether this sensitivity trade-off could be mitigated, we repeated the workflow analysis at a more conservative operating point. For DR, raising the target sensitivity from 99.0% to 99.5% reduced the per-reader sensitivity decrement relative to human-only reading from approximately 3 percentage points to about 1 percentage point, recovering most of the loss at the cost of only a marginal increase in residual review, whereas for AMD the same change substantially increased residual review with little sensitivity recovery and left the decrement statistically significant. RVO was unaffected at either operating point. The markedly low disease-specific OR for DR reflects amplification on the log-odds scale at very high baseline performance rather than a large absolute loss.

Because AI-negative images are not reviewed by an ophthalmologist, any false-negative triage decision would not be recovered by subsequent human interpretation within this workflow. Therefore, the key implementation question is whether the residual sensitivity loss is acceptable for each disease after selecting a clinically conservative operating threshold. In the primary MRMC analysis, sensitivity decreased significantly for both DR and AMD, whereas RVO sensitivity was preserved. Threshold sensitivity analysis showed that a more conservative operating point partially mitigated the sensitivity loss for DR with only a small increase in residual review, but provided limited sensitivity recovery for AMD despite a substantially larger increase in review burden. These findings suggest that threshold adjustment alone may not sufficiently resolve the safety–efficiency trade-off for all disease targets and support the prospective evaluation of additional safeguards, such as uncertainty-based referral or selective review of borderline AI-negative cases.

The post hoc, exploratory qualitative analysis of false-negative cases provides insight into the interaction between reader labeling constraints and disease-specific AI algorithms. Several AMD-labeled false negatives showed atypical or overlapping features, including myopic patchy chorioretinal atrophy, geographic atrophy, peripapillary atrophy, and extensive subretinal fibrosis. Because myopic macular degeneration was not included as a separate category and the grading protocol did not provide an “other abnormality” option, some clinically significant non-target or overlapping abnormalities may have been assigned to the closest available target category. The DR false negatives included both relatively subtle moderate nonproliferative disease and advanced or treatment-altered phenotypes, such as fibrovascular proliferation and regressed proliferative DR with laser scars. Collectively, these findings suggest that false-negative triage may arise not only from subtle lesions but also from atypical, overlapping, or treatment-modified appearances that are not fully represented by conventional disease categories. This qualitative analysis was exploratory, however, and does not establish the specific mechanisms underlying individual AI errors.

In contrast, the AI system trained to detect characteristic features of DR, RVO, and AMD correctly identified the absence of these specific features and therefore generated negative outputs. While such classifications were appropriate within the algorithm’s defined scope, this discrepancy highlights a potential limitation of disease-specific AI models in negative-screening workflows, particularly when encountering out-of-distribution retinal pathologies. Although one case of typical geographic atrophy was also missed, the majority of apparent sensitivity loss appears to be attributable to differences between pragmatic human labeling under constrained categories and the algorithm’s disease-specific feature recognition.

The reader-level analyses suggest that the effect of AI-assisted triage may not be identical across readers. Although residual workload was broadly similar within each disease, improvements in specificity and accuracy were not uniform. In AMD, for example, specificity improved for all three readers, but a significant improvement in accuracy was observed only for Reader 1, who had the lowest baseline specificity under the human-only workflow. This pattern suggests that the practical benefit of AI-assisted triage may be greater in settings where human-only reading produces more false-positive classifications. Accordingly, the effect of workflow deployment may vary across clinical settings even when the same algorithm is used, because baseline reader performance and case mix differ. AI-assisted negative-screening should therefore be viewed as a context-dependent workflow strategy rather than a uniform replacement model.

Exclusion of discordant reference cases may preferentially remove clinically ambiguous images relevant to both false-negative risk and apparent efficiency. This effect was greatest for AMD, for which 6.1%–11.6% of images were excluded and full-cohort referral burden was 19.6%, compared with 13.5%–16.8% in the primary reader-agreed sets. The primary AMD estimates should therefore be interpreted as results for reader-agreed cases rather than definitive performance in a full screening population containing ambiguous findings. Because discordant cases were excluded identically from the AI-assisted and human-only arms, the paired within-set comparison was not subject to differential exclusion between workflows. Nevertheless, the relative workflow effect may differ in an unselected screening population containing ambiguous cases.

Because the present study simulated AI-assisted triage using ophthalmologist interpretations obtained without access to AI outputs, it cannot determine how real-time AI information would influence subsequent human reading. In actual use, AI-positive referral or disease-specific AI outputs may shift clinician attention, introduce anchoring, or lead to inappropriate over-reliance or under-reliance on AI recommendations [27]. Conversely, AI-negative cases are not reviewed in this workflow, leaving no opportunity for a clinician to override a false-negative triage decision. Therefore, the diagnostic performance estimated in this study should be interpreted as a workflow-level simulation under fixed reader decisions, rather than as the prospective performance of an interactive human-AI system. These behavioral effects, including automation bias and trust calibration, should be evaluated in prospective studies incorporating real-time AI interaction [27,28].

Furthermore, future studies should prospectively evaluate this triage workflow in routine practice, including discordant and ambiguous cases excluded from the present analyses. They should measure actual reading time and downstream referral consequences, and assess how access to AI outputs influences reader behavior, including potential automation-related bias. External validation in populations with different prevalence profiles and in non-tertiary settings will also be important, because the clinical usefulness of this strategy depends on the context-specific trade-off between missed positives and workload reduction. The present workflow remains relevant to current clinical practice because most commercially deployed retinal AI systems are still primarily based on task-specific image-classification models for screening or referral decisions. Nevertheless, clinical AI frameworks are increasingly moving toward multimodal, language-aligned, and knowledge-grounded systems, as discussed by Neha et al. [29]. Bhati et al. [30], in their cross-family survey of large language model architectures, alignment methods, and benchmark performance, highlighted the importance of model alignment and evaluation for downstream AI applications. In parallel, retrieval-augmented generation frameworks, reviewed by Neha et al. [31], may enable AI systems to incorporate external clinical evidence, patient metadata, and language-based reasoning to support evidence-linked outputs, reporting, and clinician-facing explanations. In this context, the negative-screening gate evaluated here should be viewed as an image-based triage component that could potentially be integrated with language-aligned or retrieval-augmented modules, rather than as a complete decision-support system. Such hybrid systems may help improve interpretability and support the evaluation of atypical or out-of-distribution findings, but these approaches were not evaluated in the present study and require prospective validation before clinical implementation.

Several limitations should be considered. First, discordant cases were excluded from the disease-specific analyses. In the composite analysis, images with no positive label but disagreement for at least one target disease between the two non-index readers were also excluded. Therefore, the reported results were derived from a consensus-defined evaluable subset rather than the full case set and may overestimate both diagnostic performance and the apparent reduction in review workload. Second, the reported workload reduction reflects direct ophthalmologist review burden for DR, RVO, and AMD only. It should not be interpreted as actual reading time or total clinical workload reduction in routine fundus interpretation. Third, this was a retrospective simulation study conducted at a single tertiary referral center. Although this design enabled a clean paired comparison of workflow effects, it could not capture changes in reader behavior when AI outputs are actually available in practice, including possible automation-related bias toward positive classifications. Fourth, patient-level and eye-level linkage identifiers were not retained after de-identification, preventing identification of repeated images from the same patient or eye and precluding clustered or hierarchical sensitivity analyses. Therefore, the present results should be interpreted as an image-level evaluation, and variance estimates may have been affected by the absence of patient- and eye-level clustering. Fifth, generalizability may be limited by the single-center tertiary referral setting, long data collection period, and possible differences in disease prevalence, referral patterns, imaging devices, acquisition conditions, and image quality. Moreover, exclusion of discordant cases and underrepresentation of ambiguous or potentially out-of-distribution pathology may make negative-screening performance appear safer than it would be in unselected populations.

In this study, AI-assisted negative-screening triage for CFP substantially reduced the proportion of cases requiring direct ophthalmologist review across DR, RVO, and AMD, while largely preserving overall diagnostic performance. Although modest differences in sensitivity were observed in some settings, specificity generally improved. Beyond reducing workload, this approach introduces a standardized AI triage gate before human interpretation, allowing the workflow to operate at a predefined sensitivity-oriented threshold while preserving ophthalmologist review for AI-positive cases. Overall, AI-assisted triage represents a practical workflow strategy that can enhance efficiency and consistency in CFP interpretation, with its clinical utility depending on the acceptable balance between workload reduction and diagnostic trade-offs.

## Supporting information

S1 Table The standalone diagnostic performance of the AI on the pairwise reader–disease datasets. (DOCX)

S2 Table Best-case and worst-case bounds on workflow performance when discordant (excluded) cases are added back to each reader-disease evaluation set (AI operating point: target sensitivity 0.99). (DOCX)

S3 Table Per-reader, per-disease workflow performance at a more conservative operating point (target sensitivity 0.995). Human-only versus AI-assisted, on the evaluable (reader-agreement) set.
(DOCX)

S4 Table Threshold sensitivity analysis: per-reader, per-disease comparison of the human-only baseline with the AI-assisted workflow at the prespecified operating point (target sensitivity 0.99) and at a more conservative operating point (target sensitivity 0.995). (DOCX)

S5 Table Additional descriptive performance metrics (PPV, NPV, and F1-score) by target disease and reader under the human-only and AI-assisted workflows (primary operating point: target sensitivity 0.99). (DOCX)

S6 Table Confusion-matrix counts by target disease and reader under the human-only and AI-assisted workflows (primary operating point: target sensitivity 0.99). (DOCX)
