# Supplementary material for: Evaluating an AI-assisted triage workflow for retinal diseases
Source: PLOS Digit Health. 2026 Sep 15;5(9):e0001700. doi: 10.1371/journal.pdig.0001700 (PMC13577534; doi:10.1371/journal.pdig.0001700)
Supplement: Supplementary file 1 [file pdig.0001700.s001.docx]

S1 Table. The standalone diagnostic performance of the AI on the pairwise reader–disease datasets.

| **Standalone AI performance** | **Diabetic Retinopathy (DR)** | | | **Retinal Vein Occlusion (RVO)** | | | **Age-related Macular Degeneration (AMD)** | | | |
| --- | --- | --- | --- | --- | --- | --- | --- | --- | --- | --- |
| **Metric** | **Subset 1 (N = 6,767)** | **Subset 2 (N = 6,746)** | **Subset 3 (N = 6,819)** | **Subset 1 (N = 6,885)** | **Subset 2 (N = 6,862)** | **Subset 3 (N = 6,869)** | **Subset 1 (N = 6,481)** | **Subset 2 (N = 6,321)** | **Subset 3 (N = 6,101)** |  |
| AUC | 0.9966 (0.9951–0.9981) | 0.9971 (0.9959–0.9983) | 0.9968 (0.9956–0.9981) | 0.9996 (0.9994–0.9998) | 0.9996 (0.9993–0.9999) | 0.9989 (0.9980–0.9997) | 0.9946 (0.9921–0.9971) | 0.9910 (0.9876–0.9945) | 0.9900 (0.9863–0.9937) |  |
| Accuracy | 0.9855 (0.9824–0.9882) | 0.9868 (0.9838–0.9894) | 0.9845 (0.9812–0.9873) | 0.9262 (0.9198–0.9323) | 0.9284 (0.9221–0.9344) | 0.9274 (0.9210–0.9334) | 0.9279 (0.9214–0.9341) | 0.9590 (0.9538–0.9638) | 0.9523 (0.9467–0.9575) |  |
| Sensitivity | 0.9541 (0.9300–0.9718) | 0.9465 (0.9215–0.9655) | 0.9544 (0.9317–0.9712) | 1.0000 (0.9882–1.0000) | 1.0000 (0.9882–1.0000) | 0.9968 (0.9822–0.9999) | 0.9857 (0.9720–0.9938) | 0.9684 (0.9543–0.9791) | 0.9507 (0.9299–0.9667) |  |
| Specificity | 0.9877 (0.9846–0.9902) | 0.9897 (0.9869–0.9920) | 0.9867 (0.9836–0.9894) | 0.9227 (0.9160–0.9291) | 0.9250 (0.9184–0.9313) | 0.9241 (0.9174–0.9304) | 0.9225 (0.9154–0.9292) | 0.9576 (0.9519–0.9628) | 0.9525 (0.9465–0.9579) |  |
| NPV | 0.9968 (0.9951–0.9981) | 0.9962 (0.9943–0.9975) | 0.9965 (0.9947–0.9978) | 1.0000 (0.9994–1.0000) | 1.0000 (0.9994–1.0000) | 0.9998 (0.9991–1.0000) | 0.9985 (0.9971–0.9994) | 0.9949 (0.9925–0.9966) | 0.9945 (0.9921–0.9963) |  |
| PPV | 0.8421 (0.8069–0.8731) | 0.8673 (0.8341–0.8961) | 0.8456 (0.8124–0.8749) | 0.3805 (0.3471–0.4147) | 0.3878 (0.3539–0.4225) | 0.3829 (0.3492–0.4174) | 0.5460 (0.5147–0.5770) | 0.7809 (0.7548–0.8055) | 0.6809 (0.6478–0.7127) |  |
| F1-score | 0.8946 (0.8731–0.9136) | 0.9052 (0.8847–0.9232) | 0.8967 (0.8764–0.9146) | 0.5512 (0.5217–0.5805) | 0.5588 (0.5291–0.5883) | 0.5533 (0.5236–0.5827) | 0.7027 (0.6795–0.7253) | 0.8646 (0.8485–0.8796) | 0.7935 (0.7714–0.8143) |  |
| TP | 416 | 425 | 460 | 312 | 311 | 309 | 552 | 827 | 559 |  |
| FP | 78 | 65 | 84 | 508 | 491 | 498 | 459 | 232 | 262 |  |
| TN | 6,253 | 6,232 | 6,253 | 6,065 | 6,060 | 6,061 | 5,462 | 5,235 | 5,251 |  |
| FN | 20 | 24 | 22 | 0 | 0 | 1 | 8 | 27 | 29 |  |
| Values in parentheses indicate 95% confidence intervals. AUC, area under the curve; NPV, negative predictive value; PPV, positive predictive value; TP, true positive; FP, false positive; TN, true negative; FN, false negative. AUC 95% CIs were calculated using the DeLong method. | | | | | | | | | |  |
