# Supplementary material for: Evaluating an AI-assisted triage workflow for retinal diseases
Source: PLOS Digit Health. 2026 Sep 15;5(9):e0001700. doi: 10.1371/journal.pdig.0001700 (PMC13577534; doi:10.1371/journal.pdig.0001700)
Supplement: Supplementary file 2 [file pdig.0001700.s002.docx]

**S2 Table.** Best-case and worst-case bounds on workflow performance when discordant (excluded) cases are added back to each reader-disease evaluation set (AI operating point: target sensitivity 0.99).

| **Disease** | **Reader** | **Workflow** | **Evaluable (n)** | **Discordant added (n)** | **Sensitivity** | | | **Specificity** | | | **Accuracy** | | |
| --- | --- | --- | --- | --- | --- | --- | --- | --- | --- | --- | --- | --- | --- |
|  |  |  |  |  | **Main** | **Worst case** | **Best case** | **Main** | **Worst case** | **Best case** | **Main** | **Worst case** | **Best case** |
| DR | R1 | Human-only | 6,767 | 137 | 0.956 | 0.876 | 0.964 | 0.995 | 0.980 | 0.995 | 0.992 | 0.972 | 0.992 |
|  |  | AI-assisted | 6,767 | 137 | 0.922 | 0.817 | 0.934 | 0.998 | 0.985 | 0.998 | 0.993 | 0.973 | 0.993 |
|  | R2 | Human-only | 6,746 | 158 | 0.929 | 0.797 | 0.940 | 1.000 | 0.987 | 1.000 | 0.995 | 0.972 | 0.995 |
|  |  | AI-assisted | 6,746 | 158 | 0.895 | 0.751 | 0.910 | 1.000 | 0.989 | 1.000 | 0.993 | 0.970 | 0.993 |
|  | R3 | Human-only | 6,819 | 85 | 0.865 | 0.808 | 0.878 | 0.994 | 0.986 | 0.994 | 0.985 | 0.972 | 0.985 |
|  |  | AI-assisted | 6,819 | 85 | 0.834 | 0.758 | 0.846 | 0.997 | 0.991 | 0.997 | 0.985 | 0.973 | 0.986 |
| RVO | R1 | Human-only | 6,885 | 19 | 0.978 | 0.953 | 0.978 | 0.997 | 0.995 | 0.997 | 0.996 | 0.993 | 0.996 |
|  |  | AI-assisted | 6,885 | 19 | 0.978 | 0.950 | 0.978 | 0.997 | 0.996 | 0.997 | 0.997 | 0.994 | 0.997 |
|  | R2 | Human-only | 6,862 | 42 | 0.981 | 0.894 | 0.981 | 1.000 | 0.998 | 1.000 | 0.999 | 0.993 | 0.999 |
|  |  | AI-assisted | 6,862 | 42 | 0.981 | 0.892 | 0.981 | 1.000 | 0.998 | 1.000 | 0.999 | 0.993 | 0.999 |
|  | R3 | Human-only | 6,869 | 35 | 0.984 | 0.919 | 0.985 | 0.999 | 0.997 | 0.999 | 0.998 | 0.993 | 0.998 |
|  |  | AI-assisted | 6,869 | 35 | 0.984 | 0.919 | 0.985 | 0.999 | 0.997 | 0.999 | 0.998 | 0.993 | 0.998 |
| AMD | R1 | Human-only | 6,481 | 423 | 0.986 | 0.857 | 0.991 | 0.920 | 0.870 | 0.921 | 0.926 | 0.869 | 0.930 |
|  |  | AI-assisted | 6,481 | 423 | 0.971 | 0.795 | 0.981 | 0.960 | 0.914 | 0.961 | 0.961 | 0.903 | 0.964 |
|  | R2 | Human-only | 6,321 | 583 | 0.646 | 0.396 | 0.664 | 0.997 | 0.989 | 0.997 | 0.949 | 0.869 | 0.954 |
|  |  | AI-assisted | 6,321 | 583 | 0.637 | 0.385 | 0.647 | 0.999 | 0.995 | 0.999 | 0.950 | 0.870 | 0.954 |
|  | R3 | Human-only | 6,101 | 802 | 0.939 | 0.511 | 0.960 | 0.988 | 0.936 | 0.989 | 0.983 | 0.869 | 0.985 |
|  |  | AI-assisted | 6,101 | 802 | 0.925 | 0.495 | 0.950 | 0.993 | 0.943 | 0.994 | 0.987 | 0.872 | 0.988 |
| For the AMD R3 evaluation, 803 images were excluded from the primary analysis, whereas 802 were included in the discordant-case bound analysis because one excluded image lacked the evaluated-reader classification required to define workflow performance. AI, artificial intelligence; DR, diabetic retinopathy; RVO, retinal vein occlusion; AMD, age-related macular degeneration. | | | | | | | | | | | | | |
