# Supplementary material for: Evaluating an AI-assisted triage workflow for retinal diseases
Source: PLOS Digit Health. 2026 Sep 15;5(9):e0001700. doi: 10.1371/journal.pdig.0001700 (PMC13577534; doi:10.1371/journal.pdig.0001700)
Supplement: Supplementary file 3 [file pdig.0001700.s003.docx]

**S3 Table.** Per-reader, per-disease workflow performance at a more conservative operating point (target sensitivity 0.995). Human-only versus AI-assisted, on the evaluable (reader-agreement) set.

| **Disease** | **Reader** | **Residual review, % (95% CI)** | **Accuracy** | | | **Sensitivity** | | | **Specificity** | | |
| --- | --- | --- | --- | --- | --- | --- | --- | --- | --- | --- | --- |
|  |  |  | **Human-only** | **AI-assisted** | ***p*^†^** | **Human-only** | **AI-assisted** | ***p*^†^** | **Human-only** | **AI-assisted** | ***p*^†^** |
| DR | R1 | 8.2 (7.5–8.9) | 0.992  (0.990–0.994) | 0.994  (0.991–0.995) | 0.052 | 0.956 (0.933–0.974) | 0.943 (0.917–0.963) | 0.031 | 0.995 (0.993–0.996) | 0.997 (0.996–0.998) | <0.001 |
|  | R2 | 8.1 (7.4–8.8) | 0.995  (0.993–0.997) | 0.994  (0.992–0.996) | 0.031 | 0.929 (0.901–0.951) | 0.915 (0.886–0.939) | 0.031 | 1.000 (0.999–1.000) | 1.000 (0.999–1.000) | 1.000 |
|  | R3 | 8.9 (8.3–9.6) | 0.985  (0.981–0.987) | 0.986  (0.983–0.988) | 0.115 | 0.865 (0.831–0.894) | 0.853 (0.818–0.883) | 0.031 | 0.994 (0.991–0.995) | 0.996 (0.994–0.997) | <0.001 |
| RVO | R1 | 12.3 (11.5–13.1) | 0.996  (0.994–0.997) | 0.997 (0.995–0.998) | 0.062 | 0.978 (0.954–0.991) | 0.978 (0.954–0.991) | 1.000 | 0.997 (0.995–0.998) | 0.997 (0.996–0.998) | 0.062 |
|  | R2 | 12.0 (11.3–12.8) | 0.999 (0.998–1.000) | 0.999 (0.998–1.000) | 1.000 | 0.981 (0.958–0.993) | 0.981 (0.958–0.993) | 1.000 | 1.000 (0.999–1.000) | 1.000 (0.999–1.000) | 1.000 |
|  | R3 | 12.1 (11.3–12.9) | 0.998 (0.997–0.999) | 0.998 (0.997–0.999) | 1.000 | 0.984 (0.963–0.995) | 0.984 (0.963–0.995) | 1.000 | 0.999 (0.998–0.999) | 0.999 (0.998–1.000) | 1.000 |
| AMD | R1 | 21.5 (20.5–22.6) | 0.926 (0.919–0.932) | 0.946 (0.940–0.951) | <0.001 | 0.986 (0.972–0.994) | 0.973 (0.956–0.985) | 0.016 | 0.920 (0.913–0.927) | 0.943 (0.937–0.949) | <0.001 |
|  | R2 | 21.4 (20.4–22.4) | 0.949 (0.944–0.955) | 0.949 (0.944–0.955) | 1.000 | 0.646 (0.613–0.678) | 0.638 (0.605–0.670) | 0.016 | 0.997 (0.995–0.998) | 0.998 (0.996–0.999) | 0.016 |
|  | R3 | 18.3 (17.4–19.3) | 0.983 (0.980–0.986) | 0.985 (0.982–0.988) | 0.064 | 0.939 (0.916–0.957) | 0.927 (0.903–0.947) | 0.016 | 0.988 (0.985–0.991) | 0.991 (0.988–0.994) | <0.001 |
| Values in parentheses indicate 95% confidence intervals. Residual review indicates the proportion of cases requiring ophthalmologist review under the AI-assisted workflow; human-only residual review was 100% by definition. ^†^P values compare the human-only and AI-assisted workflows within each reader–disease pair and were calculated using exact McNemar tests. AI, artificial intelligence; DR, diabetic retinopathy; RVO, retinal vein occlusion; AMD, age-related macular degeneration. | | | | | | | | | | | |
