# Supplementary material for: Evaluating an AI-assisted triage workflow for retinal diseases
Source: PLOS Digit Health. 2026 Sep 15;5(9):e0001700. doi: 10.1371/journal.pdig.0001700 (PMC13577534; doi:10.1371/journal.pdig.0001700)
Supplement: Supplementary file 4 [file pdig.0001700.s004.docx]

**S4 Table.** Threshold sensitivity analysis: per-reader, per-disease comparison of the human-only baseline with the AI-assisted workflow at the prespecified operating point (target sensitivity 0.99) and at a more conservative operating point (target sensitivity 0.995).

| **Disease** | **Reader** | **Residual review (%)** | | | **Sensitivity** | | | ***p*^†^**  **(vs human-only)** | |
| --- | --- | --- | --- | --- | --- | --- | --- | --- | --- |
|  |  | **Human-only** | **AI, 0.99** | **AI, 0.995** | **Human-only** | **AI, 0.99** | **AI, 0.995** | **0.99** | **0.995** |
| DR | R1 | 100.0 | 7.3 | 8.2 | 0.956 | 0.922 | 0.943 | <0.001 | 0.031 |
|  | R2 | 100.0 | 7.3 | 8.1 | 0.929 | 0.895 | 0.915 | <0.001 | 0.031 |
|  | R3 | 100.0 | 8.0 | 8.9 | 0.865 | 0.834 | 0.853 | <0.001 | 0.031 |
| RVO | R1 | 100.0 | 11.9 | 12.3 | 0.978 | 0.978 | 0.978 | 1.000 | 1.000 |
|  | R2 | 100.0 | 11.7 | 12.0 | 0.981 | 0.981 | 0.981 | 1.000 | 1.000 |
|  | R3 | 100.0 | 11.7 | 12.1 | 0.984 | 0.984 | 0.984 | 1.000 | 1.000 |
| AMD | R1 | 100.0 | 15.6 | 21.5 | 0.986 | 0.971 | 0.973 | 0.008 | 0.016 |
|  | R2 | 100.0 | 16.8 | 21.4 | 0.646 | 0.637 | 0.638 | 0.008 | 0.016 |
|  | R3 | 100.0 | 13.5 | 18.3 | 0.939 | 0.925 | 0.927 | 0.008 | 0.016 |
| ^†^P values compare sensitivity between the human-only workflow and the AI-assisted workflow at each operating point within each reader–disease pair and were calculated using exact McNemar tests. AI, artificial intelligence; DR, diabetic retinopathy; RVO, retinal vein occlusion; AMD, age-related macular degeneration. | | | | | | | | | |
