# Supplementary material for: Evaluating an AI-assisted triage workflow for retinal diseases
Source: PLOS Digit Health. 2026 Sep 15;5(9):e0001700. doi: 10.1371/journal.pdig.0001700 (PMC13577534; doi:10.1371/journal.pdig.0001700)
Supplement: Supplementary file 5 [file pdig.0001700.s005.docx]

**Table S5.** Additional descriptive performance metrics (PPV, NPV, and F1-score) by target disease and reader under the human-only and AI-assisted workflows (primary operating point: target sensitivity 0.99).

| **Disease** | **Reader** | **Human-only** | | | **AI-assisted** | | |
| --- | --- | --- | --- | --- | --- | --- | --- |
|  |  | **PPV**  **(95% CI)** | **NPV**  **(95% CI)** | **F1-score**  **(95% CI)** | **PPV (95% CI)** | **NPV (95% CI)** | **F1-score (95% CI)** |
| DR | R1 | 0.925  (0.896–0.947) | 0.997  (0.995–0.998) | 0.940  (0.923–0.955) | 0.969 (0.947–0.983) | 0.995 (0.993–0.996) | 0.945 (0.927–0.959) |
|  | R2 | 1.000  (0.991–1.000) | 0.995  (0.993–0.997) | 0.963  (0.948–0.975) | 1.000 (0.991–1.000) | 0.993 (0.990–0.995) | 0.945 (0.927–0.959) |
|  | R3 | 0.913  (0.883–0.937) | 0.990  (0.987–0.992) | 0.888  (0.866–0.908) | 0.955 (0.930–0.973) | 0.988 (0.985–0.990) | 0.890 (0.868–0.910) |
| RVO | R1 | 0.933  (0.900–0.957) | 0.999  (0.998–1.000) | 0.955  (0.936–0.969) | 0.947 (0.917–0.969) | 0.999 (0.998–1.000) | 0.962 (0.944–0.976) |
|  | R2 | 1.000  (0.988–1.000) | 0.999  (0.998–1.000) | 0.990  (0.979–0.996) | 1.000 (0.988–1.000) | 0.999 (0.998–1.000) | 0.990 (0.979–0.996) |
|  | R3 | 0.974  (0.950–0.989) | 0.999  (0.998–1.000) | 0.979  (0.965–0.989) | 0.978 (0.954–0.991) | 0.999 (0.998–1.000) | 0.981 (0.967–0.990) |
| AMD | R1 | 0.539  (0.507–0.569) | 0.999  (0.997–0.999) | 0.697  (0.673–0.719) | 0.699 (0.666–0.731) | 0.997 (0.995–0.998) | 0.813 (0.791–0.834) |
|  | R2 | 0.967  (0.949–0.980) | 0.948  (0.941–0.953) | 0.775  (0.752–0.796) | 0.987 (0.974–0.995) | 0.946 (0.940–0.952) | 0.774 (0.752–0.796) |
|  | R3 | 0.895  (0.868–0.918) | 0.993  (0.991–0.995) | 0.916  (0.899–0.931) | 0.936 (0.913–0.955) | 0.992 (0.989–0.994) | 0.931 (0.915–0.945) |
| AI, artificial intelligence; DR, diabetic retinopathy; RVO, retinal vein occlusion; AMD, age-related macular degeneration; NPV, negative predictive value; PPV, positive predictive value; CI, confidence interval. | | | | | | | |
