# Supplementary material for: Evaluating an AI-assisted triage workflow for retinal diseases
Source: PLOS Digit Health. 2026 Sep 15;5(9):e0001700. doi: 10.1371/journal.pdig.0001700 (PMC13577534; doi:10.1371/journal.pdig.0001700)
Supplement: Supplementary file 6 [file pdig.0001700.s006.docx]

**Table S6.** Confusion-matrix counts by target disease and reader under the human-only and AI-assisted workflows (primary operating point: target sensitivity 0.99).

| **Disease** | **Reader** | **Human-only** | | | | **AI-assisted** | | | |
| --- | --- | --- | --- | --- | --- | --- | --- | --- | --- |
|  |  | **TP** | **FP** | **TN** | **FN** | **TP** | **FP** | **TN** | **FN** |
| DR | R1 | 417 | 34 | 6297 | 19 | 402 | 13 | 6318 | 34 |
|  | R2 | 417 | 0 | 6297 | 32 | 402 | 0 | 6297 | 47 |
|  | R3 | 417 | 40 | 6297 | 65 | 402 | 19 | 6318 | 80 |
| RVO | R1 | 305 | 22 | 6551 | 7 | 305 | 17 | 6556 | 7 |
|  | R2 | 305 | 0 | 6551 | 6 | 305 | 0 | 6551 | 6 |
|  | R3 | 305 | 8 | 6551 | 5 | 305 | 7 | 6552 | 5 |
| AMD | R1 | 552 | 473 | 5448 | 8 | 544 | 234 | 5687 | 16 |
|  | R2 | 552 | 19 | 5448 | 302 | 544 | 7 | 5460 | 310 |
|  | R3 | 552 | 65 | 5448 | 36 | 544 | 37 | 5476 | 44 |
| AI, artificial intelligence; DR, diabetic retinopathy; RVO, retinal vein occlusion; AMD, age-related macular degeneration; TP, true positive; FP, false positive; TN, true negative; FN, false negative. | | | | | | | | | |
